# Salmon in Combination with High Glycemic Index Carbohydrates Increases Diet-Induced Thermogenesis Compared with Salmon with Low Glycemic Index Carbohydrates–An Acute Randomized Cross-Over Meal Test Study

**DOI:** 10.3390/nu11020365

**Published:** 2019-02-10

**Authors:** Lone V. Nielsen, Signe Nyby, Lars Klingenberg, Christian Ritz, Ulrik K. Sundekilde, Hanne C. Bertram, Margriet S. Westerterp-Plantenga, Bjørn Liaset, Karsten Kristiansen, Lise Madsen, Anne Raben

**Affiliations:** 1 Department of Nutrition, Exercise and Sports, University of Copenhagen, 1958 Frederiksberg C, Denmark; lvn@nexs.ku.dk (L.V.N.); sherby@live.dk (S.N.); lakl@nexs.ku.dk (L.K.); ritz@nexs.ku.dk (C.R.); 2 Department of Biology, University of Copenhagen, 2100 København Ø, Denmark; KK@bio.ku.dk (K.K.); Lise.Madsen@hi.no (L.M.); 3 Department of Food Science, Aarhus University, 5792 Aarslev, Denmark; uksundekilde@food.au.dk (U.K.S.); hannec.bertram@food.au.dk (H.C.B.); 4 NUTRIM School of Nutrition and Translational Research in Metabolism, Maastricht University, 6229 ER Maastricht, The Netherlands; m.westerterp@maastrichtuniversity.nl; 5 Institute of Marine Research, 5005 Bergen, Norway; Bjorn.Liaset@hi.no

**Keywords:** protein, fish, meat, appetite, amino acids, ghrelin, glucose, energy expenditure, energy intake, mixed meals

## Abstract

The study investigated the acute effects of meals containing either salmon or veal in combination with carbohydrates with high or low glycemic index (GI) on diet-induced thermogenesis (DIT) (primary endpoint), appetite sensations, and energy intake (EI). Twenty-five overweight men and women ingested four iso-caloric test meals: salmon with mashed potatoes (high GI) (SM), salmon with wholegrain pasta (low GI) (SP), veal with mashed potatoes (VM) and veal with wholegrain pasta (VP). Energy expenditure was measured in the fasting state and six times postprandially for 25 min with 5-min breaks between each measurement. Appetite sensations were measured every 30 min. Blood samples, from arterialized venous blood, were drawn every 20 min until an *ad libitum* buffet-style lunch was served 3.5 h later. DIT was 40% higher after the SM meal compared to the SP meal (*p* = 0.002). Prospective food consumption was lower after the SM meal compared with the VP meal (*p* = 0.01). There were no differences in satiety, hunger, fullness, or *ad libitum* EI between the test meals (all *p* > 0.05). In conclusion, salmon with high GI carbohydrates increased DIT compared to salmon with low GI carbohydrates. This indicates that DIT is sensitive to the GI of the carbohydrates after intake of salmon but not veal.

## 1. Introduction

High protein diets increase diet-induced thermogenesis (DIT) and satiety compared with low protein diets [1,2,3,4]. As proteins have unique characteristics depending on the amino acid composition and absorption rate, it has been speculated whether proteins from different sources affect DIT and appetite differently. Studies on the impact of different protein sources on DIT are sparse, whereas the impact on appetite is more investigated [5,6,7,8,9,10,11,12]. However, the lack of adequate standardization and the use of different types of protein meals complicate the interpretation of these studies. 

Health authorities recommend the general population to consume seafood, particularly oily fish, in order to reduce the risk of cardiovascular diseases [13]. Fish and seafood are the main dietary contributors to marine n-3 polyunsaturated fatty acids (PUFA), but fish also contains vitamin D, selenium, iodine, and high-quality proteins, all being crucial nutrients [13]. Protein from different fish sources has been shown to increase satiety and reduce *ad libitum* energy intake (EI) compared with animal proteins (beef, chicken, egg, or turkey) [5,6,7,8]. However, the majority of these studies have methodological shortcomings such as a varying protein content between the tested protein sources [6], texture differences and lack of information regarding weight of the test meals [5], or use of liquid test meals, which may not reflect the effect of foods in solid form [7,14]. 

The glycemic index (GI) is a ranking of carbohydrates according to their effect on blood glucose responses [15]. The majority of the human studies which have investigated the effect of GI on DIT have not shown any effect [16,17,18,19,20]. However, compared to a diet with fish oil in combination with high GI carbohydrates, mice fed a diet with fish oil in combination with low GI carbohydrates had increased oxygen consumption and carbon dioxide production in the fed state [21]. This indicates that the type of carbohydrate in meals with fish oil could influence the thermic response of the meal. The acute effect of mixed meals with low and high GI carbohydrates on appetite and EI has been investigated in numerous human studies [22,23,24]. However, the evidence for a reduced appetite and EI after meals with low compared with high GI carbohydrates is not convincing. 

The objective of the current study was to investigate the acute effects of meals containing salmon versus veal in combination with carbohydrates with high or low GI on DIT (primary endpoint), appetite and EI, as well as postprandial responses of ghrelin, plasma amino acids, glucose, and insulin. We hypothesized that salmon in combination with low GI carbohydrates would increase DIT compared to salmon in combination with high GI carbohydrates or veal in combination with carbohydrates with low or high GI.

## 2. Materials and Methods

### 2.1. Study Design

This randomized, 4-way, crossover study was conducted at the Department of Nutrition, Exercise and Sport, University of Copenhagen, from September 2014 to March 2015. The study was carried out in accordance with the Declaration of Helsinki and all subjects gave their written informed consent for inclusion before they participated in the study. The protocol was approved by the Municipal Ethical Committee of the Capital Region, Denmark (journal number H-1-2014-038) and the study was registered at clinicaltrials.gov (NCT02770833). 

### 2.2. Experimental Protocol

On the test days, which were conducted with a separation of minimum one week, the participants arrived in a fasting state at 7:30 AM. The participants were then required to try to void urine, and body weight was measured (Lindeltronic 8000, Samhall lavi AB, Kristianstad, Sweden). Hereafter, participants laid down in a supine position and the non-dominant hand was placed in a heat box. Blood pressure and temperature were measured after 15 min of rest and an intravenous catheter was inserted into the back of the heated hand. At 8:15 AM, appetite and gastrointestinal feelings were registered and a fasting blood sample was drawn [25]. Then, resting metabolic rate (RMR) was measured with a ventilated hood system (Jaeger Oxycon PRO; Viasys Healthcare GmbH, Höchberg, Germany). At 8:55 AM, the participants received one of the four test meals. They were instructed to eat the test meal at a constant pace and to distribute the consumption over a period of 15 min. Hereafter, appetite sensations and general palatability of the test meal were assessed with visual analog scales (VAS). The following three hours, respiratory gas exchange was measured six times for 25 min with 5-min breaks with a ventilated hood system. Appetite sensations were assessed with VAS every half hour after the test meal, and blood samples were drawn every 20 min until 12:30 PM, where gastrointestinal experiences were also assessed. Then, the participants were required to empty their bladder, and the urine was collected for nitrogen analysis. The participants were, afterwards, served an *ad libitum* buffet-style lunch. A table of the measurements and time points can be found in Table S1.

### 2.3. Subjects

Healthy overweight men and women were recruited from the Copenhagen area through advertisements in newspapers, the web page www.forsøgsperson.dk, and the website of the Department of Nutrition Exercise and Sports. To be included in the study, participants had to have a body mass index (BMI) between 25.0 and 30.0 kg/m^2^. This was chosen as overweight individuals often wish to lose weight. Furthermore, the participants had to be non-smoking, between 18 and 50 years of age, with a fasting blood glucose level below 5.9 mmol/L. Exclusion criteria included: chronic diseases, use of prescription medication that has the potential to affect body weight or glucose metabolism, psychoactive medication, epileptic medication or weight loss medications, self-reported eating disorders or irregular eating schedule (e.g., skipping breakfast), food allergies, substance abuse, vigorous physical activity of more than 10 h/week, alcohol intake above the recommendations from the Danish Health Authority (7 or 14 units per week for women and men, respectively), daily caffeine intake above 300 mg, night- or shift work, blood donation less than 1 month before study commencement and during the study period, and simultaneous participation in other clinical studies. Pregnant or breastfeeding women or women who intended to become pregnant during the study period, menopausal women, and women with an irregular menstrual cycle were furthermore excluded. 

### 2.4. Standardization

The participants were required and instructed to eat a standardized 3500 kJ dinner, provided as frozen product, between 7:00 PM and 8:00 PM the evening before the test days, the dinner consisted of a chicken paprika dish served with rice and orange juice: (16.6 E% protein, 50.1 E% carbohydrate and 33.3 E% fat, 4.9 g fiber/meal). From 8:00 PM the participants were required to fast. However, consumption of 500 mL water was allowed during fasting, though maximum 250 mL in the morning of the test day. The participants were not allowed to do vigorous physical activity, take any medicine, or to drink alcohol 48 h before the test days. On the test days, participants arrived by transport requiring a minimum of physical activity (i.e., by car, bus or train). They were required to use the same means of transportation on all four test days. 

### 2.5. Test Meals

The test meals were: patties of salmon served with mashed potatoes (SM), patties of salmon served with wholegrain pasta (SP), patties of veal served with mashed potatoes (VM), and patties of veal served with wholegrain pasta (VP). Additionally, the test meals were served with tomato sauce and water. Based on data from the Danish dietary software program Dankost 3000^®^ (Dansk Catering Center, Copenhagen, Denmark), the test meals were iso-caloric ~ 2011 kJ/meal and had similar macronutrient distribution (approximately 25.5 E% protein, 40.5 E% carbohydrate, 34.0 E% fat), energy density, and fiber content (~ 4.9 g), see Table 1. The wholegrain pasta was characterized as low GI and the mashed potatoes as high GI [26]. 

The concentrations of individual amino acids in the diets were analyzed on the ACQUITY UPLC^®^ System (Waters, Milford, MA, USA), except for tryptophan. The methods have been described previously [27]. Based on the analyses of individual amino acids in the test meals, the SM, SP, VM, and VP meals contained a total of 25.6 g, 25.4 g, 29.5 g, and 30.1 g amino acids, respectively, see Table 2.

### 2.6. Energy Expenditure and Substrate Oxidation

Energy expenditure (EE) and substrate oxidation were measured with a ventilated hood system (Jaeger Oxycon PRO; Viasys Healthcare GmbH, Höchberg, Germany). The precision of the system was validated by a weekly alcohol-burning test. EE and oxidation of carbohydrate, fat, and protein were calculated from the gas exchange and the urinary nitrogen measurements using constants of Elia and Livesey [28]. DIT was calculated as area under the EE curve above fasting (RMR) level (kJ/min* min), the last 20 min of the 25 min measurements of EE were used for the calculation.

### 2.7. Visual Analog Scales

Appetite sensations and evaluations of the meals were assessed by VAS with a length of 100 mm [25]. At each end of the line, the most extreme rating was expressed [25]. Participants answered questions regarding satiety, hunger, fullness, prospective food consumption (PFC), and desire to eat something salty, sweet, meat/fish, or fat-rich. The test meals and the *ad libitum* buffet-style lunch were rated with regard to visual appearance, odor, palatability, off taste and general appearance. Zero corresponded to the most positive rating and 100 to the most negative rating in the evaluation of the meals. The participants completed eight yes/no questions regarding gastrointestinal symptoms (bloating, diarrhea, rumbling, throat burn, flatulence, nausea, acid reflux, and stomach pain). The questionnaires on gastrointestinal symptoms were filled in before the test meal was ingested, and at 110 and 200 min after ingestion of the test meal. If the participants answered yes to a gastrointestinal symptom, they had to register the intensity on a VAS of 100 mm in length. The VAS questionnaires were answered on an electronic tablet-based VAS (eVAS). The eVAS system was set up on an HP Slate 2 tablet using Acqui version 1 (xyzt, Copenhagen, Denmark). Appetite ratings with eVAS has previously been validated and found comparable to the pen-and-paper method [12].

### 2.8. Ad Libitum Buffet Style Lunch

The *ad libitum* buffet style lunch consisted of a variety of cold and hot foods, and represented a typical Danish lunch. The participants were instructed to eat at a constant pace and to stop eating when they felt pleasantly satiated. All foods were weighted to the nearest gram, by an experienced food technician, before and after the meal.

### 2.9. Biochemical Analyses

All blood samples were drawn by the heated-hand box method [29]. A catheter was inserted to a vein on the back on the hand, and the hand was then placed in a cavity where heated air circulated to warm the hand to 50 °C.

Blood for analyses of plasma lactate and glucose was drawn into tubes prepared with sodium fluoride-oxalate. Blood collected for analyses of serum C-peptide and insulin was drawn into serum clot activator tubes. Blood for analysis of plasma triglycerides (TAG) was drawn into tubes prepared with EDTA while blood for analysis of plasma ghrelin levels was drawn into EDTA-prepared tubes containing aprotinin. Immediately after the blood samples were drawn, blood for analyses of glucose, lactate, TAG, and ghrelin was centrifuged (2500 × g for 10 min at 4 °C). Blood for analyses of insulin and C-peptide was allowed to coagulate for 20 min before centrifugation. Afterwards, all samples were frozen and stored at −80 °C until they were analyzed. Plasma lactate, glucose and TAG were analyzed on an ABX Pentra 400, HORIBA, CA, USA (plasma glucose: intra CV %: 1.4, inter CV %: 2.5; plasma lactate: intra CV %: 0.2, inter CV %: 0.9, plasma TAG: intra CV %: 1.5, inter CV % 2.0). Serum insulin and C-peptide were analyzed on an Immulite 1000, Siemens, Erlangen, Germany (serum insulin: intra CV %: 4.2, inter CV %: 4.2; serum C-peptide: intra CV %: 2.5, inter CV %: 1.8). Total plasma ghrelin was analyzed with an enzyme-linked immunosorbent assay produced by EMD Millipore, MA, USA (intra CV %: 3.8, inter CV %: 8.9). 

To calculate protein oxidation, all urine produced by the participants during the measurements of EE on each test day was collected and weighed, and a 10 mL sample was used for the analysis of nitrogen. Urinary nitrogen was measured on an Elementar VarioMax CN analyzer, Elementar, Langenselbold, Germany (intra CV %: 2.0 %, inter CV%: 3.4). 

Plasma amino acids were analyzed in a subsample of five subjects (all sampling points), which were randomly chosen among the subjects. The small subsample was due to financial constraints. Blood samples for analyses of plasma amino acids were drawn into heparin-prepared tubes, and plasma amino acids were quantified by nuclear magnetic resonance (NMR) spectroscopy. Prior to NMR spectroscopy, the 300µL of plasma sample was filtered using Amicon Ultra 0.5 mL 3 kDa (Millipore, MA, USA) spin filters at 14,000 g for 1 h at 4 °C. 200 µL of filtered sample was added 70 µL D_2_O and 330 µL phosphate buffer also containing 3-(Trimethylsilyl)-1-propanesulfonic acid-d_6_ sodium salt (DSS; Sigma-Aldrich, MO, USA) and formate (Sigma-Aldrich, MO, USA). Final concentration of buffer was 50 mM Na_2_HPO_4_, 0.25 mM DSS, and 117 µM formate. ^1^H NMR spectroscopy was performed at 298 K on a Bruker Avance III 600 spectrometer, operating at a ^1^H frequency of 600.13 MHz, and equipped with a 5-mm ^1^H TXI probe (Bruker BioSpin, MA, USA). The sample sequence was randomized prior to acquisition and standard one-dimensional spectra were acquired using noesypr1d pulse sequence with water presaturation during relaxation delay and mixing time. Relaxation delay was 5 s, mixing time was 0.1 s and a total of 64 scans were collected into 32,768 data points spanning a spectral width of 12.02 ppm. All ^1^H NMR spectra were referenced to the DSS signal at 0 ppm. The data was multiplied by a 0.3 Hz line-broadening function prior to Fourier transformation. The proton NMR spectra were phase- and baseline corrected manually using Topspin 3.2 (Bruker BioSpin, MA, USA). Amino acids were quantified using Chenomx NMR Suite 8.1.2 (Chenomx Incorporated, Edmonton, Canada).

### 2.10. Blinding and Randomization

The participants were randomly allocated to a combination of the four test meals. The randomization, which was conducted by the study coordinator, was stratified between gender, i.e., two separate randomization lists of the meal sequences were generated using an online randomization program [30]: one to allocate men and one to allocate women. It was not possible to blind the study, as the smell and appearance of the four test meals could not be concealed. 

### 2.11. Sample Size

The sample size calculation was based on results from a previous study [31]. Thus, 20 subjects would give a statistical power of 90% to detect a 35 kJ difference in DIT with a within-subject SD of 32 kJ at a 2-sided 5% significance level. 

### 2.12. Statistical Analysis

Baseline data are presented as mean ± standard derivation (SD). Models for summary measures (DIT, iAOC [incremental area over the curve], iAUC [incremental area under the curve], *ad libitum* EI, time to peak, and the evaluation of the test meals) included meal as a main effect and subject as a random effect, and were adjusted for sex, age, BMI, and visit number. In case a significant meal effect was found, model-based pairwise comparisons, adjusted for multiple testing, were used to identify differences between test meals. DIT, iAUC, and iAOC were calculated using the trapezoidal rule. Time to peak was determined as the time point where the maximum response was observed. Models to investigate differences in satiety, hunger, fullness, or PFC, both as summary measures and repeated measures, were additionally adjusted for palatability of the test meals. Repeated measurements were analyzed with linear mixed models. The models included a meal-time interaction and overall subject and within-visit subject differences were included as random effects. The models were adjusted for age, sex, visit number, BMI, and fasting value on the test day. Serial correlation between repeated measurements for the same subject within each visit was modeled, assuming a spatial Gaussian correlation structure (exponentially decreasing correlation over time). Time points with differences between meals were identified with model-based pairwise comparisons, adjusted for multiple testing. Multiplicity adjustment of *P* values was based on the single-step method [32]. 

For all models, assumption of normality and homogeneity of variance were evaluated graphically using residual plots and normal probability plots. Fisher’s exact test was used to analyze if there were differences in the occurrence of gastrointestinal side effects. 

Results are presented as mean ± standard error (SE) or mean differences between meals ± SE unless otherwise stated. Graphs are based on unweighted averages. *P* values < 0.05 were considered significant. All statistical analyses were conducted in R version 3.1.2 (R Core Team, 2016, Vienna, Austria).

## 3. Results

### 3.1. Subjects

Forty subjects attended a screening visit, 15 of these did not meet the inclusion criteria. Thus, 25 subjects, 12 men and 13 women, were randomized to the study (Table 3). 

**Figure 1:**
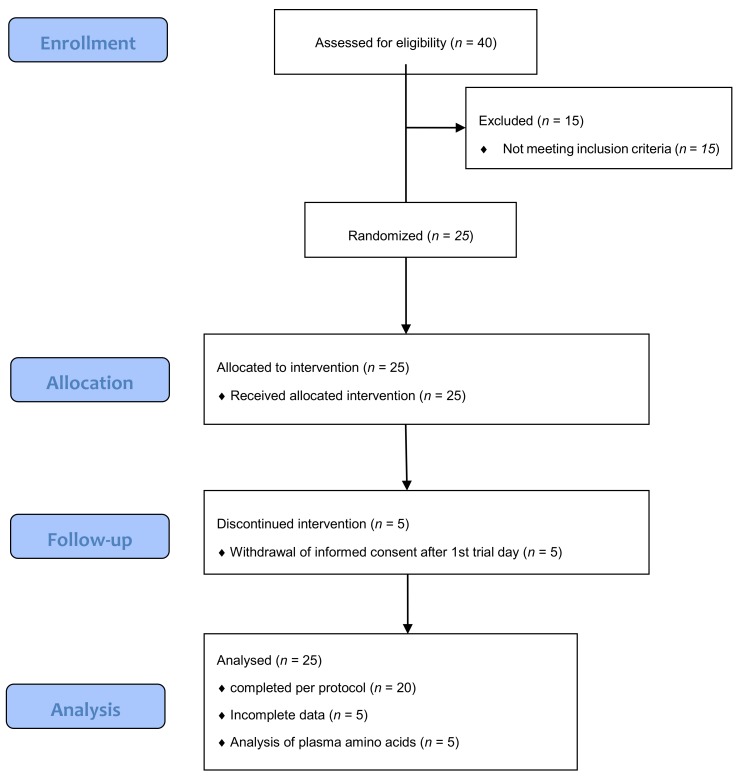
Participant flowchart.

Five subjects dropped out after the first test day. Twenty subjects completed all four test days, see (Figure 1). 

### 3.2. DIT and Substrate Oxidation

**Figure 2:**
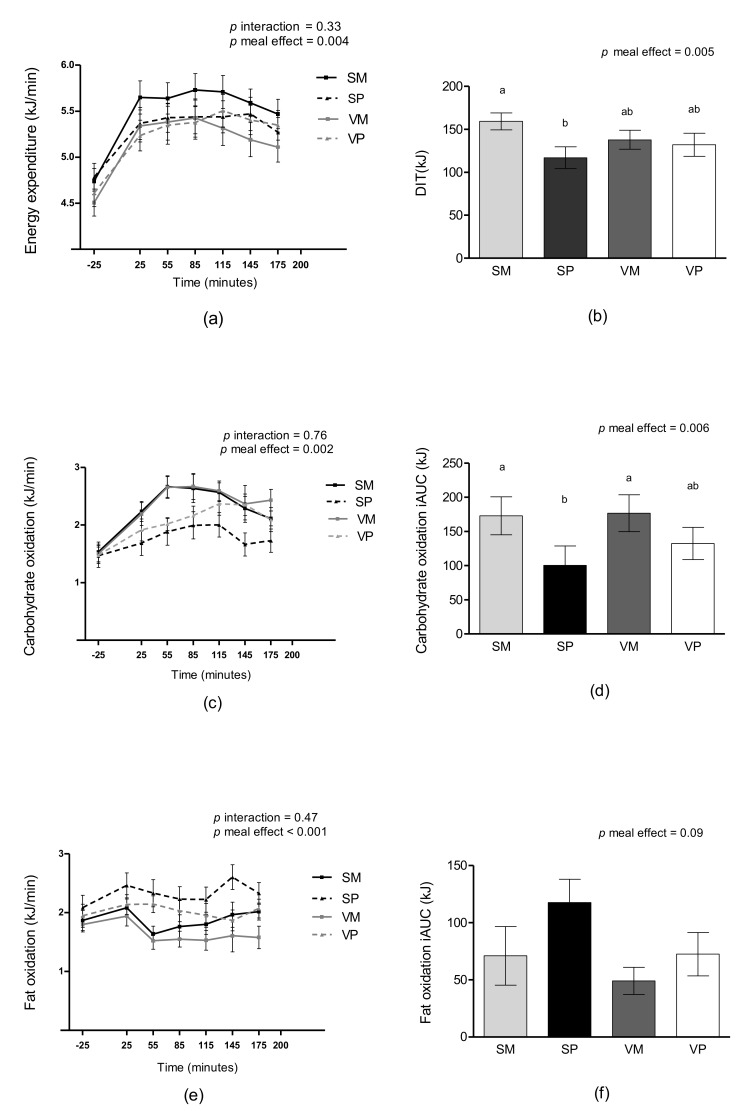
Mean unadjusted measures of EE (**a**), DIT (**b**), carbohydrate oxidation (**c**), carbohydrate oxidation iAUC (**d**), fat oxidation (**e**), and fat oxidation iAUC (**f**) after intake of four different test meals: SM, SP, VM, and VP. Data are presented as means ± standard error of mean (SEM), *n* = 25. Measurements over time were analyzed as repeated measures including a meal-time interaction using linear mixed models, post hoc comparisons were single-step adjusted. iAUC was analyzed using a linear mixed model including meal as a fixed effect, means not sharing a common letter differ. DIT, diet-induced thermogenesis; EE, energy expenditure; iAUC, incremental area under the curve; SM, salmon and mashed potatoes; SP, salmon and wholegrain pasta; VM, veal and mashed potatoes; VP, veal and wholegrain pasta.

A main effect of meal was found for DIT (*p* = 0.005), see Figure 2b. DIT was 40% higher after the SM meal compared to the SP meal (*p* = 0.002). No interaction between meal and time was found for the postprandial increase in EE (*p* = 0.33). However, a meal effect was found (*p* = 0.004). The SM meal resulted in an increased postprandial EE compared to the SP meal (mean difference between the SM and the SP meal: 0.24 ± 0.07 kJ/min, *p* = 0.001) and the VP meal (mean difference between the SM meal and the VP meal: 0.18 ± 0.07 kJ/min, *p* = 0.04), see Figure 2a.

No meal-time interaction was found for postprandial fat oxidation or carbohydrate oxidation (*p =* 0.47 and *p =* 0.76, respectively). However, a main effect of meal was found for both parameters (*p* < 0.001 and *p* = 0.002, respectively). Carbohydrate oxidation was increased after the SM meal compared to the SP meal (*p* = 0.03) and after the VM meal compared to the SP meal (*p* = 0.004) and the VP meal (*p* = 0.049). When analyzed as iAUC a higher carbohydrate oxidation was found after the SM and VM meals compared to the SP meal (all *p* < 0.05), see Figure 2c and Figure 2d. 

Fat oxidation was higher after the SP meal than after SM, VM, and VP meals (*p* = 0.001, *p* < 0.001, and *p* = 0.038, respectively). Additionally, fat oxidation was higher after the SM meal compared to the VM meal (*p* = 0.036) and after the VP meal compared with the VM meal (*p*= 0.001). When fat oxidation was analyzed as iAUC no differences were found between meals, see Figure 2e and Figure 2f. There were no differences in protein oxidation between the test meals (*p =* 0.52).

### 3.3. Appetite Sensations

**Figure 3:**
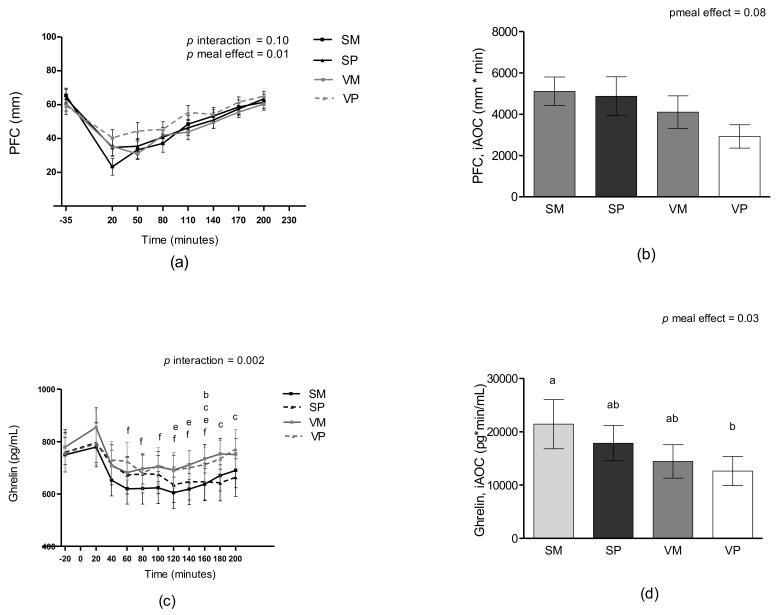
Mean unadjusted measures of PFC (**a**), iAOC PFC (**b**), plasma ghrelin (**c**) and iAOC ghrelin (**d**) after intake of four different test meals: SM, SP, VM, and VP. Data are presented as means ± SEM, *n* = 25. Measurements over time were analyzed as repeated measures including a meal-time interaction using linear mixed models. ^b^ difference between the VM and SM meals (*P* < 0.05), ^c^ difference between the VM and SP meals (*P* < 0.05), ^e^ difference between the SP and VP meals (*P* < 0.05), ^f^ difference between the SM and VP meals (*P* < 0.05). iAOC was analyzed using linear mixed models including meal as a fixed effect, means not sharing a common letter differ. PFC, prospective food consumption; iAOC, incremental area over the curve; iAUC, incremental area under the curve; SM, salmon and mashed potatoes; SP, salmon and wholegrain pasta; VM, veal and mashed potatoes; VP, veal and wholegrain pasta.

There were no interactions between meal and time in any of the appetite parameters (satiety, hunger, fullness, or PFC) (all *p* > 0.05). A meal effect was found for PFC (*p* = 0.012), see Figure 3a. Here, the VP meal resulted in a higher PFC compared to the SM meal (8.7 ± 2.9 mm, *p* = 0.022). There were no differences in satiety, hunger, or fullness. The results were similar when the appetite parameters were adjusted for palatability of the test meals or overall appearance. There were no differences between test meals when satiety, fullness, hunger, or PFC were analyzed as iAUC or iAOC (all *p* > 0.05). 

No interactions between meal and time or meal effects were observed for the desire to eat meat or fish, or for the specific taste of sweet or salt. However, a meal effect was found for the desire for something fat rich, which was lower after the SM meal compared to the SP meal (12.5 ± 3.8 mm, *p* = 0.005). 

### 3.4. Ad Libitum Energy Intake

There were no differences between the four test meals in *ad libitum* EI or the macronutrient distribution of the ingested food at the buffet style lunch (all *p* > 0.05) (Table 4). 

### 3.5. Ghrelin

There was a significant meal-time interaction for plasma ghrelin (*p* = 0.002). Pairwise comparisons showed that plasma ghrelin levels were generally more suppressed after the salmon meals compared to the veal meals, see Figure 3c. When ghrelin was analyzed as iAOC a meal effect was found (*p* = 0.03), iAOC was higher after the SM meal compared with the VP meal (*p =* 0.02). 

### 3.6. Glucose and Insulin

**Figure 4:**
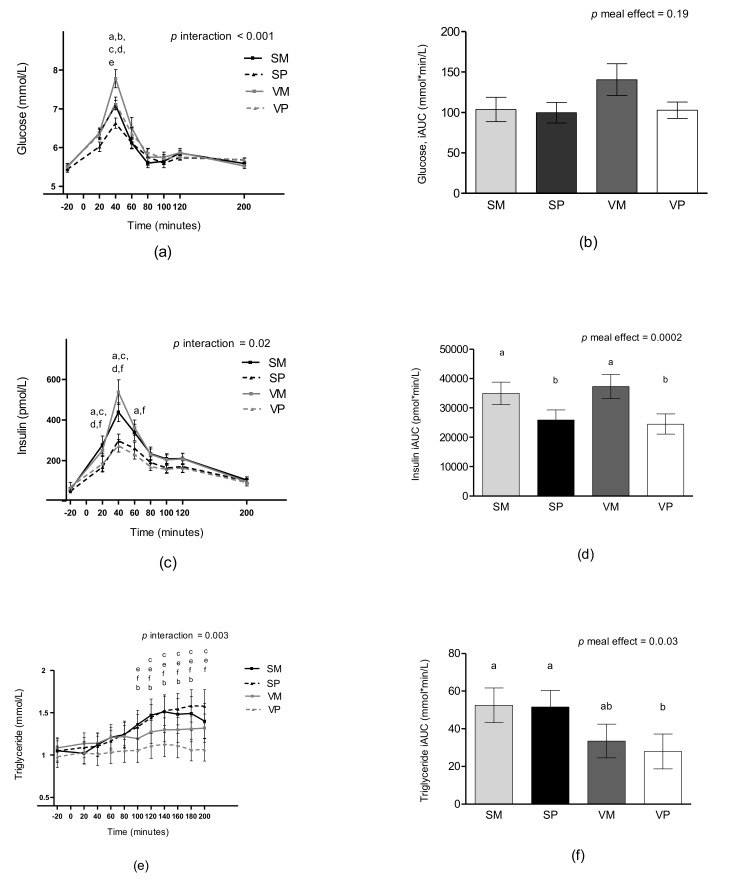
Mean unadjusted concentrations of plasma glucose (**a**), plasma glucose iAUC (**b**), serum insulin (**c**), serum insulin iAUC (**d**), plasma triglyceride (**e**), and plasma triglyceride iAUC (**f**) after intake of four different test meals: SM, SP, VM, and VP. Data are presented as means ± SEM, *n* = 25. Measurements over time were analyzed as repeated measures including a meal-time interaction using linear mixed models, post hoc comparisons were single-step adjusted. Meal-time interactions were found for glucose, insulin and triglyceride. ^a^ difference between the VM and VP meals (*p* < 0.05), ^b^ difference between the VM and SM meals (*p* < 0.05), ^c^ difference between the VM and SP meals (*p* < 0.05), ^d^ difference between the SM and SP meals (*p* < 0.05), ^e^ difference between the SP and VP meals (*p* < 0.05), ^f^ difference between the SM and VP meals (*p* < 0.05). iAUC was analyzed using linear mixed models including meal as a fixed effect, means not sharing a common letter differ. iAUC, incremental area under the curve; SM, salmon and mashed potatoes; SP, salmon and pasta; VM, veal and mashed potatoes; VP, veal and pasta.

A meal-time interaction was found for postprandial glucose concentrations (*p* < 0.001). Thus, the glucose peak 40 min after the test meal was significantly higher after the meals with veal compared to the meals with salmon in combination with the same type of carbohydrate (mean difference between the VM and the SM meal: 0.67 ± 0.16 mmol/L *p* < 0.001, mean difference between the VP and the SP meal: 0.44 ± 0.16 *p* = 0.033). The VM meal resulted in the highest glucose peak, while the glucose peak was lowest after the SP meal (all *p* for comparisons between meals < 0.05). The SM meal and the VP meal induced a similar glucose peak (*p* > 0.999). When glucose concentrations were analyzed as iAUC no differences were found between test meals (Figure 4a and Figure 4b). 

A meal-time interaction was observed for insulin. The meals with mashed potatoes induced higher insulin responses at 20 and 40 min compared with the meals with pasta (all *p* < 0.05). When insulin was analyzed as iAUC a meal effect was found (*p =* 0.0002), the meals with mashed potatoes induced a higher insulin response compared with the meals with pasta (all *p* < 0.05), see Figure 4c and Figure 4d. 

A meal-time interaction was also found for plasma lactate and serum C-peptide (*p* = 0.01 and *p* = 0.003, respectively). Differences between the test meals again depended on the type of carbohydrate (Figure S1).

### 3.7. Triglycerides

A meal-time interaction was found for TAG (*p =* 0.003). From 100 to 200 min, plasma TAG concentrations were generally higher after the salmon meals compared to the veal meals, see Figure 4e. When TAG was analyzes as iAUC a meal effect was found (*p =* 0.003), showing that the SM meal and the SP meal induced higher responses compared with the VP meal (*p =* 0.01 and *p* = 0.01, respectively).

### 3.8. Plasma Amino Acids

**Figure 5:**
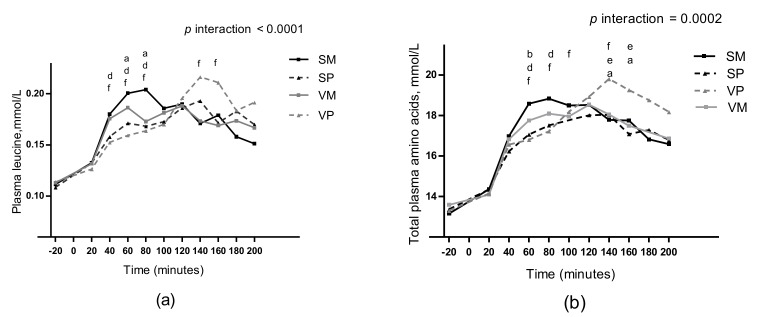
Mean unadjusted changes in plasma leucine (**a**) and total plasma amino acids (**b**), after intake of four different test meals: SM, SP, VM, and VP. Data are presented as means ± SEM, *n* = 5. Data were analyzed as repeated measures including a meal-time interaction using linear mixed models. Post hoc comparisons were single-step adjusted. ^a^ difference between the VM and VP meals (*p* < 0.05), ^b^ difference between the VM and SM meals (*p* < 0.05), ^d^ difference between the SM and SP meals (*p* < 0.05), ^e^ difference between the SP and VP meals (*p* < 0.05), ^f^ difference between the SM and VP meals (*p* < 0.05). SM, salmon and mashed potatoes; SP, salmon and pasta; VM, veal and mashed potatoes; VP, veal and pasta.

Postprandial plasma responses of leucine and total amino acids are shown in Figure 5. A meal-time interaction was found for the postprandial increase in plasma concentrations of total amino acids, leucine, tyrosine, asparagine, glycine, histidine, isoleucine, lysine, methionine, phenylalanine, threonine and valine (all *p* < 0.05), whereas meal effects were found for alanine and arginine (Table S2). A meal effect was found for time to peak of total plasma amino acid concentration (*p* = 0.006). Time to peak was 82 ± 37 min after the SM meal, 128 ± 33 min after the SP meal, 93 ± 32 min after the VM meal, and 132 ± 46 min after the VP meal. The total amino acid concentration peaked earlier after the SM meal compared with the SP and VP meals (*p* = 0.008 and *p* = 0.03, respectively), no other differences were observed in time to peak (all *p* > 0.05), see Figure 5b. 

### 3.9. Evaluations of the Test Meals

The SM meal resulted in a lower ratings of visual appearance and general appearance compared to the VP meal (*p* = 0.004 and *p* = 0.04, respectively). Moreover, the SM meal resulted in a lower rating of visual appearance compared to the VM meal (*p* = 0.03), (Table 5). There were no differences in the evaluation of palatability, off taste, or smell between the four test meals (all *p* > 0.05). 

### 3.10. Wel-Bleing and Gastrointestinal Side Effects

There were no differences in the evaluation of well-being between the test meals (*p* = 0.35) or in the incidence of gastrointestinal side effects (all *p* > 0.05).

## 4. Discussion

The current study showed a higher DIT after a meal with salmon and high GI carbohydrates compared to a meal with salmon and low GI carbohydrates. Thus, we were unable to confirm our hypothesis that the meal with salmon in combination with low GI carbohydrates would increase DIT compared to the salmon meal with high GI carbohydrates and the veal meals with carbohydrates with low or high GI. The higher DIT after the salmon meal with high GI carbohydrates compared with the salmon meal with low GI carbohydrates, but not after the veal meal with high GI carbohydrates compared to the veal meal with low GI carbohydrates, indicates that the GI is not responsible for the observed difference per se. The difference must, therefore, be a combined effect of salmon and the high GI carbohydrates. We observed that the total plasma amino acid concentration peaked significantly faster after the salmon meal with high GI carbohydrates compared with the salmon and veal meals in combination with low GI carbohydrates. This indicates more rapid digestion of the proteins and/or uptake of amino acids in the salmon meal with high GI carbohydrates. Rapidly digested proteins have been shown to increase DIT compared with more slowly digested proteins, probably mediated by an increased postprandial protein synthesis [33,34]. Additionally, the salmon meal with high GI carbohydrates resulted in a higher plasma concentration of the branched-chain amino acid leucine compared to the meals with salmon or veal in combination with low GI carbohydrates. High plasma concentrations of leucine have been shown to enhance the rate of postprandial protein synthesis, and leucine has, in addition, been shown to be more thermogenic than other amino acids in rats [35,36]. Thus, differences in the rate of postprandial protein synthesis after the four meals may in part explain the difference in DIT observed in the present study. 

We found differences in carbohydrate oxidation between the meals. The differences within the salmon and veal meals were dependent on the GI of the carbohydrates with the meals, with a high GI resulting in increased carbohydrate oxidation. Conversely, fat oxidation was higher after ingestion of the meals with low GI. Fat oxidation was, furthermore, higher after the salmon meals compared with the veal meals in combination with the same GI of the accompanying carbohydrates. It has been hypothesized that high-GI meals compared with low-GI meals increase carbohydrate oxidation and reduce fat oxidation [16]. Our results confirm this hypothesis, which is also supported by some previous studies [20,37]. However, it has not been shown consistently, and several studies have not found any differences in substrate oxidation between meals with low or high GI carbohydrates [16,17,18,19]. 

After both salmon meals, an increase in postprandial TAG concentration was found. This is in agreement with results from previous acute studies which, opposed to studies of longer durations, have shown higher TAG responses after meals rich in n-3 PUFAs compared to meals low in n-3 PUFAs [38,39,40]. Robertson et al. observed, additionally, a more rapid gastric emptying after a meal rich in n-3 PUFAs compared with meals rich in saturated fatty acids, monounsaturated fatty acids, or n-6 PUFAs [38]. Thus, it is possible that the elevated postprandial TAG response after the salmon meals could be a result of a more rapid gastric emptying. 

In the current study, we did not observe differences in satiety, hunger, fullness, or *ad libitum* EI between the test meals. However, PFC was lower after the salmon meal with high GI carbohydrates compared with the veal meal with low GI carbohydrates. Protein from different fish species such as ling fish, tuna, cod, and *antarcticus* has previously been shown to increase satiety compared with protein from animal sources [5,7,8]. Uhe et al. found increased satiety after consumption of fish (*mustelus antarcticus*) compared to beef and chicken [5]. The authors observed that plasma amino acids took significantly longer time to peak after consumption of fish. They suggest that this may have been a result of slower digestion, slower gastric emptying, or slower absorption after fish, and that this could be related to the beneficial effect of fish on appetite regulation. In the current study, we investigated time to peak of the total concentration of plasma amino acids, and found a faster time to peak after the salmon meal with high GI carbohydrates compared with the meals with salmon or veal and low GI carbohydrates. In contrast to the study by Uhe et al. PFC was lowest after the meal with the fastest peak of total amino acids, which does not comply with the suggested decrease in appetite after slowly absorbed proteins [5]. Furthermore, differences in time to peak between the test meals in this present study did not influence satiety, hunger, fullness, or EI and were not dependent on protein source per se, but rather a combined effect of protein source and type of carbohydrate. 

We found a difference in ghrelin levels between the meals. The ghrelin levels were generally suppressed after the salmon meals compared to the veal meals. Plasma ghrelin levels have, in obese women, previously been demonstrated to be reduced after a high-fat meal with PUFA compared to a high-fat meal with saturated fatty acids [41]. Thus, the differences in ghrelin levels between the salmon and veal meals could be a result of the differences in fatty acid composition.

We found that differences in serum insulin seemed to reflect the GI of the carbohydrates regardless of protein source. However, serum insulin concentrations did not reflect the differences in plasma glucose concentrations as the salmon meals induced lower glucose peaks compared to the veal meals in combination with the same GI of the accompanying carbohydrates. Thus, our data demonstrate that the combination of foods in mixed meals influences postprandial glucose regulation. This might be of importance in subjects with impaired ability to control blood glucose as larger fluctuations in blood glucose are unwanted. The long chain n-3 PUFA could be speculated to influence the postprandial glucose response. Some authors have demonstrated that the addition of unsaturated fat to carbohydrates resulted in a lower glucose response compared to the addition of saturated fat [42,43], although this has not been shown consistently [44,45,46]. Different proteins may also affect the glycemic response differently. It is known that some amino acids stimulate insulin secretion and promote glucose uptake [47]. However, our results suggest an insulin-independent effect of salmon on plasma glucose. It could, additionally, be speculated that impaired glucose absorption from the intestines or increased storage of the glucose in the liver as glycogen could be responsible for the difference in postprandial glucose response. However, the higher DIT and carbohydrate oxidation after the SM meal (and partly the VM meal) indicates that glucose is taken up in tissues and used as energy substrate through glycolysis. 

### 4.1. Strengths and Limitations

The current study has both strengths and limitations, which should be taken into account. The strengths included the randomized crossover study design, our thorough standardization, and the use of the protein sources in real meals with low and high GI, which made it possible to investigate the influence of the accompanying carbohydrates. Based on the Danish dietary software program Dankost 3000^®^ (Dansk Catering Center, Copenhagen, Denmark), the test meals were iso-caloric and macronutrient-balanced, and had the same weight and fiber content. However, laboratory analysis of the amino acid composition of the meals revealed the meals with salmon had an approximately 4.5 g lower protein content than the calculated content. The protein content in the veal meals was consistent with the calculated value. Discrepancies may be a result of the analytical method used to determine protein content in foods. Data in Dankost 3000^®^ are based on data from the Danish food composition database. Here, the protein content is based on an analysis of total nitrogen content; the nitrogen content is then multiplied by a factor of 6.25 to calculate protein content [48]. However, salmon and seafood have, in general, a relatively high amount of non-protein nitrogen-compounds compared with land animals [49]. Consequently, the protein content of seafood may be estimated to be higher than the actual amount [49]. This is problematic and a weakness of the present study. However, it nonetheless emphasizes the importance of analyzing the actual meal composition. Further weaknesses of the current study includes the small subsample of five participants for the analyses of plasma amino acids and the limitations of the NMR method for amino acid analyses, which was unable to detect cysteine and tryptophan as they were below limit of detection. Consequently, total plasma amino acid concentrations were based on the sum of plasma amino acids without these two amino acids. 

### 4.2. Conclusions

In conclusion, salmon with high GI carbohydrates increased DIT compared to macronutrient-balanced meals with salmon and low GI carbohydrates, whereas no difference was found in comparisons with meals with veal and high or low GI carbohydrates. This finding indicates a combined effect of salmon and high GI carbohydrate on DIT. We propose that an earlier plasma amino acid peak after the meal with salmon and high GI carbohydrates compared with the meal with salmon and low GI carbohydrates could be responsible for the higher DIT. Protein from salmon and veal did not influence appetite sensations or energy intake differently. However, the appetite response may be influenced by the combination of protein source and GI, as a difference was seen in prospective food consumption between the meal with salmon and high GI carbohydrates compared with the meal with veal and low GI carbohydrates.

## Figures and Tables

**Table 1:** Recipes and nutritional content of the four test meals.

	SM	SP	VM	VP
Salmon filet, g	108.8	108.8	-	-
Wholegrain pasta, g	-	45.2	-	44
Veal, g	-	-	108.8	108.8
Mashed Potato Powder, g	48.0	-	46.8	-
Tomato puree concentrate, g	17.6	17.6	17.6	17.6
Onion, g	22.4	22.4	22.4	22.4
Table salt, g	2.4	2.4	2.4	2.4
Water, g	28.8	28.8	28.8	28.8
Whole egg, g	9.6	9.6	9.6	9.6
Onion, g	9.6	9.6	9.6	9.6
Cream 38% fat, g	11.2	11.2	11.2	11.2
Breadcrumbs, g	9.6	9.6	9.6	9.6
Water with meal, g	216	219	217	220
Energy kJ	2012	2011	2012	2010
Total weight (g)	484	484	484	484
Energy density (kJ/g)	4.16	4.15	4.16	4.15
Protein %E	25.1	25.7	25.1	25.7
Protein, g	29.8	30.4	29.8	30.3
Fat %E	34.3	33.3	35.0	34.0
Fat, g	18.6	18.1	19.0	18.5
Carbohydrate %E	40.6	41.1	39.8	40.3
Carbohydrate, g	45.8	46.2	44.9	45.3
Fiber, g	4.8	5.0	4.7	4.9
Saturated fat, g	6.6	5.7	9.1	8.2
Mono unsaturated fat, g	5.4	5.4	7.3	7.3
Poly unsaturated fat, g	4.4	4.4	0.9	0.9
n-3 fatty acids	3.6	3.6	0.2	0.2
n-6 fatty acids	0.8	0.8	0.7	0.7
GI	85	40	85	40
GL	35	16	38	18

SM, salmon and mashed potatoes; SP, salmon and pasta; VM, veal and mashed potatoes; VP, veal and pasta. E%, energy percentage; GI, glycemic index; GL, glycemic load.

**Table 2:** Amino acids composition of the four test meals ^1^.

	SM	SP	VM	VP
	g/meal			
Histidine	0.68	0.68	0.90	0.89
Taurine	0.03	0.04	0.07	0.07
Serine	1.11	1.14	1.22	1.28
Arginine	1.52	1.48	1.81	1.84
Glycine	1.21	1.18	1.53	1.64
Aspartic acid	3.05	2.41	3.25	2.72
Glutamic acid	4.10	4.81	5.10	5.83
Threonine	1.20	1.13	1.28	1.25
Alanine	1.46	1.41	1.67	1.69
Lysine	2.28	2.02	2.51	2.30
Tyrosine	0.94	0.89	0.97	0.96
Methionine	0.72	0.69	0.70	0.72
Valine	1.44	1.37	1.50	1.47
Isoleucine	1.15	1.10	1.26	1.24
Leucine	2.05	2.04	2.33	2.37
Phenylalanine	1.19	1.23	1.29	1.34
Proline	1.07	1.36	1.43	1.75
Hydroxyproline	0.06	0.05	0.26	0.29
Tryptophan	0.31	0.37	0.37	0.42
Total	25.59	25.39	29.46	30.08

^1^ Asparagine and glutamine are not included as they are hydrolyzed to aspartic acid and glutamic acid, respectively. The concentration of cysteine was not assessed. SM, salmon and mashed potatoes; SP, salmon and pasta; VM, veal and mashed potatoes; VP, veal and pasta.

**Table 3:** Baseline characteristics of the 25 overweight men and women ^1^.

Characteristics	Values
Age, y	28.8 ± 7.6
Weight, kg	83.3 ± 9.2
Height, m	174 ± 7
BMI, kg/m^2^	27.5 ± 1.5
Systolic blood pressure, mmHg	119 ± 9
Diastolic blood pressure, mmHg	73 ± 6
Fasting glucose, mmol/L	5.1 ± 0.4

^1^ Data are presented as mean ± SD.

**Table 4:** *Ad libitum* energy intake after the four different test meals ^1^.

	SM	SP	VM	VP
Total energy intake, kJ	4911± 559	4975 ± 556	5059 ± 545	5059 ± 535
Protein, kJ	735 ± 110	812 ± 109	808 ± 107	808 ± 104
Carbohydrates, kJ	2301 ± 222	2275 ± 220	2320 ± 214	2184 ± 207
Fat, kJ	1863 ± 286	1881 ± 284	1919 ± 278	2061 ± 272

^1^ Data are presented as means ± SE. Data was analyzed using linear mixed models with meal as fixed effect, adjusted for sex, age, BMI, and visit number. There were no differences between test meals in total energy intake or macronutrient distribution. SM, salmon and mashed potatoes; SP, salmon and wholegrain pasta; VM, veal and mashed potatoes; VP, veal and wholegrain pasta.

**Table 5:** Palatability evaluations of the four test meals ^1^.

	SM	SP	VM	VP
General appearance (mm)	63.6 ± 6.4 ^a^	59.8 ± 6.3	53.5 ± 6.0	50.5 ± 5.5 ^a^
Look (mm)	78.1 ± 6.8 ^ab^	72.4 ± 6.7	63.9 ± 6.4 ^b^	60.5 ± 5.9 ^a^
Off taste (mm)	29.0 ± 8.1	32.5 ± 8.1	25.9 ± 7.7	24.8 ± 7.0
Smell (mm)	52.0 ± 6.8	42.0 ± 6.9	43.7 ± 6.5	40.1 ± 5.9
Palatability (mm)	43.7 ± 6.4	45.6 ± 6.4	36.1 ± 6.1	33.7 ± 5.5

^1^ Data are presented as means ± SE. Data was analyzed using linear mixed models with meal as fixed effect, adjusted for sex, age, BMI, and visit number. 100 mm represent the most negative evaluation. Values sharing a common letter are significantly different. mm, millimeters; SM, salmon and mashed potatoes; SP, salmon and pasta; VM, veal and mashed potatoes; VP, veal and pasta.

## Supplementary Materials

The following are available online at http://www.mdpi.com/2072-6643/11/2/365/s1, Figure S1: Mean unadjusted 200 min changes in serum C-peptide and plasma lactate, Table S1: Measurements in the study, Table S2: Differences between the four test meals in plasma amino acids concentrations.
