# Supplementary material for: Salmon in Combination with High Glycemic Index Carbohydrates Increases Diet-Induced Thermogenesis Compared with Salmon with Low Glycemic Index Carbohydrates–An Acute Randomized Cross-Over Meal Test Study
Source: Nutrients. 2019 Feb 10;11(2):365. doi: 10.3390/nu11020365 (PMC6412964; doi:10.3390/nu11020365)
Supplement: Supplementary file 1 [file nutrients-11-00365-s001.zip › TABLE S1.docx]

**Online Supporting Material**

**Table S1.** Measurements in the study

^1^ The numbers in the grey boxes represent the time intervals (in minutes) where energy expenditure was measured.

|  |  | Time from baseline, minutes | | | | | | | | | | | | | | | | | | | | | | | |  |
| --- | --- | --- | --- | --- | --- | --- | --- | --- | --- | --- | --- | --- | --- | --- | --- | --- | --- | --- | --- | --- | --- | --- | --- | --- | --- | --- |
|  | -85 | -35 | -25 | -20 | 0 | 20 | 25 | 40 | 50 | 55 | 60 | 80 | 85 | 100 | 110 | 115 | 120 | 140 | 145 | 160 | 170 | 175 | 180 | 200 | 215 | ~245 |
| Test meal |  |  |  |  | x |  |  |  |  |  |  |  |  |  |  |  |  |  |  |  |  |  |  |  |  |  |
| Energy expenditure^1^ |  |  | -25 - 0 | |  |  | 25 – 50 | |  | 55 – 80 | |  | 85 – 110 | |  | 115 - 140 | |  | 145 - 170 | |  | 175 - 200 | |  |  |  |
| Blood samples |  |  |  | x |  | x |  | x |  |  | x | x |  | x |  |  | x | x |  | x |  |  | x | x |  |  |
| VAS |  | x |  |  |  | x |  |  | x |  |  | x |  |  | x |  |  | x |  |  | x |  |  | x |  | x |
| *Ad libitum* buffet |  |  |  |  |  |  |  |  |  |  |  |  |  |  |  |  |  |  |  |  |  |  |  |  | x |  |
| Urine collection |  |  |  |  |  |  |  |  |  |  |  |  |  |  |  |  |  |  |  |  |  |  |  |  |  |  |
