# Supplementary material for: Salmon in Combination with High Glycemic Index Carbohydrates Increases Diet-Induced Thermogenesis Compared with Salmon with Low Glycemic Index Carbohydrates–An Acute Randomized Cross-Over Meal Test Study
Source: Nutrients. 2019 Feb 10;11(2):365. doi: 10.3390/nu11020365 (PMC6412964; doi:10.3390/nu11020365)
Supplement: Supplementary file 1 [file nutrients-11-00365-s001.zip › TABLE S2.docx]

|  | **Time-meal interaction** | **Meal effect** | **20 min** | **40 min** | **60 min** | **80 min** | **100 min** | **120 min** | **140 min** | **160 min** | **180 min** | **200 min** |
| --- | --- | --- | --- | --- | --- | --- | --- | --- | --- | --- | --- | --- |
|  | ***P*** | ***P*** | **diff** | **diff** | **diff** | **diff** | **diff** | **diff** | **diff** | **diff** | **diff** | **diff** |
| Total AA | 0.0002 | *-* | - | - | SM > SP  SM > VM  SM > VP | SM > SP  SM > VP | SM > SP |  | VP > SM  VP > SP  VP > VM | VP > SP  VP > VM |  |  |
| Leucine | < 0.0001 | - | - | SM > SP  SM > VP | SM > SP  SM > VP  VM > VP | SM > SP  SM > VM  SM > VP | - | - | - | VP > SM | - | VP > SM |
| Tyrosine | 0.0002 | - | SM > VP | SM > VP | SM > SP,  SM > VP  SP > VP  VM > VP | SM > VP,  SP > VP  VM > VP | SM > SP  SM > VP | SM > VM  SM > VP | - | - | - | - |
| Alanine | 0.6928 | 0.0106  SM > SP  VP > SP | - | - | - | - | - | - | - | - | - | - |
| Arginine | 0.0351 | 0.0056  SM > SP,  SM > VM,  SM > VP | - | - | - | - | - | - | - | - | - | - |
| Aspargine | 0.03 | - | - | SM > VP  VM > SP  VM > VP | SM > SP  SM > VP  VM > SP  VM > VP | SM > VP  VM > VP | SM > SP  SM > VP  VM > SP  VM > VP | SM > SP  SM > VP | - | SM > VP | - | - |
| Glutamate | 0.2997 | 0.2922 | - | - | - | - | - | - | - | - | - | - |
| Glutamine | 0.4115 | 0.1184 | - | - | - | - | - | - | - | - | - | - |
| Glycine | 0.0227 | - | VP > SP  VP > VM | - | - | - | VP > SP | VP > SM  VP > SP | VP > SM,  VP > SP  VP > VM, | VP > SM  VP > SP  VM > SP  VP > VM | VP > SM  VP > SP,  VM > SP | VP > SM  VP > SP  VM > SP |
| Histidine | 0.004 | - | - | - | - | - | - | VP > VM | VP > SM  VP > SP  VP > VM | VP > SM  VP > SP  VP > VM | VP > SM  VP > SP | - |
| Isoleucine | <0.0001 | - | - | SM > SP  SM > VP  VM > VP | SM > SP  SM > VM  SM > VP  SP > VP  VM > VP | SM > SP  SM > VM  SM > VP  VM > VP | SM > SP  SM > VM  SM > VP | SM > SP  SM > VM  SM > VP | - | - | - | - |
| Lysine | 0.005 | - | - | - | SM > SP  SM > VM  SM > VP | SM > SP  SM > VP | - | - | - | - | VP > SP  VP > VM | - |
| Methionine | <0.0001 | - | - | - | SM > SP  SM > VM  SM > VP | SM > SP  SM > VP  VM > VP | - | - | - | - | VP > SP  VP > VM | - |
|  | **Time-meal interaction** | **Meal effect** | **20 min** | **40min** | **60min** | **80min** | **100min** | **120min** | **140min** | **160min** | **180min** | **200min** |
|  | ***P*** | ***P*** | **diff** | **diff** | **diff** | **diff** | **diff** | **diff** | **diff** | **diff** | **diff** | **diff** |
| Phenylalanine | <0.001 | - | - | SM > SP  SM > VP | SM > SP  SM > VM  VM > VP | SM > SP  SM > VM  SM > VP | SM > VM | - | - | - | - | SP > SM  SP > VM |
| Proline | 0.6646 | 0.1141 |  |  |  |  |  |  |  |  |  |  |
| Serine | 0.1141 | 0.0916 |  |  |  |  |  |  |  |  |  |  |
| Threonine | 0.007 | - | - | SM > VP  VM > VP | - | SM > VP | SM > SP  VM > SP | - | - | SM > SP  VP > SP | - | - |
| Valine | <0.001 | - | - | SM > SP  SM > VP  VM > SP  VM > VP | SM > SP  SM > VP | SM > SP  SM > VP | SM > SP  SM > VM | SM > SP | - | - | - | - |

**SM: salmon and mashed potatoes, SP: salmon and pasta, VM: veal and mashed potatoes; VP: veal and pasta; AA: amino ac**
