# Supplementary material for: Salmon in Combination with High Glycemic Index Carbohydrates Increases Diet-Induced Thermogenesis Compared with Salmon with Low Glycemic Index Carbohydrates–An Acute Randomized Cross-Over Meal Test Study
Source: Nutrients. 2019 Feb 10;11(2):365. doi: 10.3390/nu11020365 (PMC6412964; doi:10.3390/nu11020365)
Supplement: Supplementary file 1 [file nutrients-11-00365-s001.zip › Figure S1 .docx]

**Online Supporting Material**

**Figure S1.** Mean unadjusted 200 minutes changes in serum C-peptide (a) and plasma lactate (b) in overweight men and women after intake of the four different test meals: SM, SP, VM, and VP. Data were log transformed to obtain normality and are presented as mean ± SEM, n=25. Concentrations over time were analyzed by repeated measures. Post-hoc model-based pairwise comparisons were adjusted for multiple testing. Time-meal interactions were found for C-peptide and lactate. ^a^ difference between the VM and VP meals (*P* < 0.05), ^c^ difference between the VM and SP meals (*P* < 0.05), ^d^ difference between the SM and SP meals (*P* < 0.05), ^f^ difference between the SM and VP meals (*P* < 0.05). SM, salmon and mashed potatoes; SP, salmon and pasta; VM, veal and mashed potatoes; VP, veal and pasta.
